# Clinical and epidemiological profile of congenital hyperinsulinism in Brazil

**DOI:** 10.3389/fendo.2025.1547855

**Published:** 2025-04-09

**Authors:** Raphael D. Liberatore Junior, Anna L. Marques, Laura L. Dos Santos, Thais M. Luciano

**Affiliations:** ^1^ Departamento de Pediatria, Faculdade de Medicina de Ribeirão Preto, Universidade de São Paulo, Ribeirão Preto, Brazil; ^2^ Pediatric Endocrinology Section, Pediatric Department, Ribeirão Preto Medical School, University of São Paulo, Ribeirão Preto, Brazil

**Keywords:** hypoglycemia, congenital hyperinsulinsm, children, newborn, hyperinsulinism hypoglycemia

## Abstract

**Introduction:**

Congenital hyperinsulinism is the most common cause of persistent hypoglycemia in children, often leading to severe neurological complications. Objective: This study aimed to describe the clinical and epidemiological profile of CHI in Brazil.

**Methods:**

A cross- sectional study was conducted with caregivers of CHI patients affiliated with the Associação do Hiperinsulinismo Congênito do Brasil. Data were collected via a structured questionnaire adapted from the HI Global Registry, covering clinical presentation, diagnostic pathways, genetic findings, and treatment strategies.

**Results:**

Caregivers of 68 patients participated. Symptoms appeared before six months of age in 60% of cases, but 35.5% initially received incorrect diagnoses. Genetic testing was performed in 43 patients, but pathogenic variants were identified in only 7 cases, while the majority (31) was not aware of their results. Diazoxide was the most used medication, though 13% required pancreatectomy. Developmental delays were reported in 44% of cases.

**Conclusions:**

Delayed diagnosis and limited access to genetic testing and specialized treatments remain significant barriers in Brazil. This study underscores the need for improved awareness, early recognition strategies, and expanded access to genetic and therapeutic resources to optimize patient outcomes.

## Introduction

Congenital hyperinsulinism (CHI) encompasses both transient and persistent forms of hyperinsulinism. While transient hyperinsulinism is commonly associated with perinatal stress and metabolic adaptation issues, persistent hyperinsulinism is often caused by genetic variants and is a major cause of hypoglycemia in infancy, characterized by excessive insulin secretion and persistent hypoketotic hypoglycemia. This dysregulation results in potentially life-threatening hypoglycemia, which, if prolonged, can lead to severe neurological complications or even death, particularly in neonates and infants (1–3).

Persistent CHI is primarily caused by pathogenic variants in genes that regulate the pathways involved in insulin secretion by pancreatic beta cells. Among these, *ABCC8* and *KCNJ11*, which encode the ATP-sensitive potassium (K_ATP) channel subunits, are the most frequently affected genes. Variants in these two genes account for 36–70% of cases. Pathogenic variants in other genes have also been associated with CHI (4, 5).

Additionally, CHI can be part of genetic syndromes, such as Beckwith-Wiedemann syndrome (BWS), which is caused by genetic or epigenetic changes in the imprinted 11p15.5 region, including paternal uniparental disomy (*UPD11p*), alterations in methylation patterns, or pathogenic variants in *CDKN1C*, sometimes in combination with paternally inherited recessive variants of *ABCC8* or *KCNJ11*. CHI is also associated with Kabuki syndrome, caused by *KMT2D* and *KDM6A* mutations, usually in a mosaic pattern. This genetic heterogeneity contributes to distinct clinical phenotypes of the disease (1, 6, 7).

CHI is histologically classified into diffuse, focal, and atypical forms, a distinction that has direct implications for prognosis and therapeutic strategies (7).

The global incidence and prevalence of CHI remain poorly characterized, and reliable estimates are still limited (8). The estimated prevalence is approximately 3 in 100,000 live births but can reach 37.4 per 100,000 in countries with high consanguinity rates (9, 10). Previous studies in Brazil have highlighted the importance of population-specific genetic variants, reinforcing the need for further research to enhance diagnostic strategies and treatment protocols tailored to local populations (7).

Studies indicate that without timely and effective management, severe hypoglycemia in CHI can cause irreversible neurological damage, including seizures, developmental delay, and cognitive impairment (7). The first-line treatment for CHI is diazoxide (1). However, a significant subset of patients is unresponsive to diazoxide or experiences intolerable side effects, necessitating second-line therapy. In such cases, somatostatin analogs (SSA), such as octreotide and lanreotide, are commonly used (1). Unfortunately, SSA efficacy varies, and their use is often limited by side effects, including gastrointestinal disturbances, biliary sludge, and potential impairment of growth hormone secretion (1).

In refractory cases, particularly diffuse CHI, pancreatectomy is often necessary. However, the procedure carries significant risks, including exocrine pancreatic insufficiency, persistent hypoglycemia, and diabetes mellitus. The incidence of post- pancreatectomy diabetes varies depending on the follow-up period, with studies reporting rates of up to 69% in the short term and nearly 100% after 10–20 years (2, 7, 11). Studies show that even after resection of 95–98% of pancreatic tissue, hypoglycemia persists in approximately one-third of cases, emphasizing the need for improved therapeutic strategies (7). Long-term complications include obesity, tall stature, and neurodevelopmental impairment, with reports indicating developmental delay in over half of operated patients (7). Given these challenges, the decision for surgical intervention must be carefully weighed against alternative medical treatments (7).

This study aims to delineate the clinical and epidemiological profile of CHI in Brazil, focusing on initial symptoms, demographic data, diagnostic processes, and treatment methods. This research seeks to enhance our understanding of CHI, thereby improving early diagnosis and intervention.

## Methods

This descriptive, cross-sectional study utilized convenience sampling. It was conducted in collaboration with the only national association dedicated to connecting patients and families affected by CHI in Brazil, the Associação do Hiperinsulinismo Congênito, to whom we express our gratitude for their collaboration. This association plays a crucial role in actively identifying cases and facilitating communication among affected families. Their primary outreach occurs through social media platforms, where they share information about the disease and offer support to families. This nonprofit community operates on a voluntary basis, relying on proactive engagement from caregivers of children diagnosed with CHI to reach and support those in need.

The studies included children who had already been diagnosed with CHI based on clinical, laboratory, and genetic criteria and were under medical follow-up, with their families being members of the CHI association.

Data collection involved a questionnaire adapted into Portuguese from the international registry form used by Congenital Hyperinsulinism International—the Global Registry. This registry, the first of its kind globally, gathers direct patient-reported information and includes questions addressing the clinical and epidemiological aspects of the disease (12). The questionnaire was translated and modified to suit the Brazilian context, ensuring cultural and contextual relevance.

Initial contact with potential participants was made via digital invitations sent to all members of the association. Those who consented were interviewed via videoconference. Data collection was based solely on caregiver reports. No medical records were reviewed, and physicians were not contacted.

The study was approved by the Ethics Committee of the Ribeirão Preto Medical School University Hospital (approval number: 11631912.1.0000.5440) and was conducted in accordance with the ethical principles of the Declaration of Helsinki, as well as local legislation and institutional requirements. To ensure confidentiality, all questionnaires were stored using unique identification codes, with only two researchers having access to the data.

Written informed consent for participation in this study was obtained from the participants’ legal guardians. Collected data were analyzed using summary statistics appropriate to the nature of each variable.

## Results

**Figure 1:**
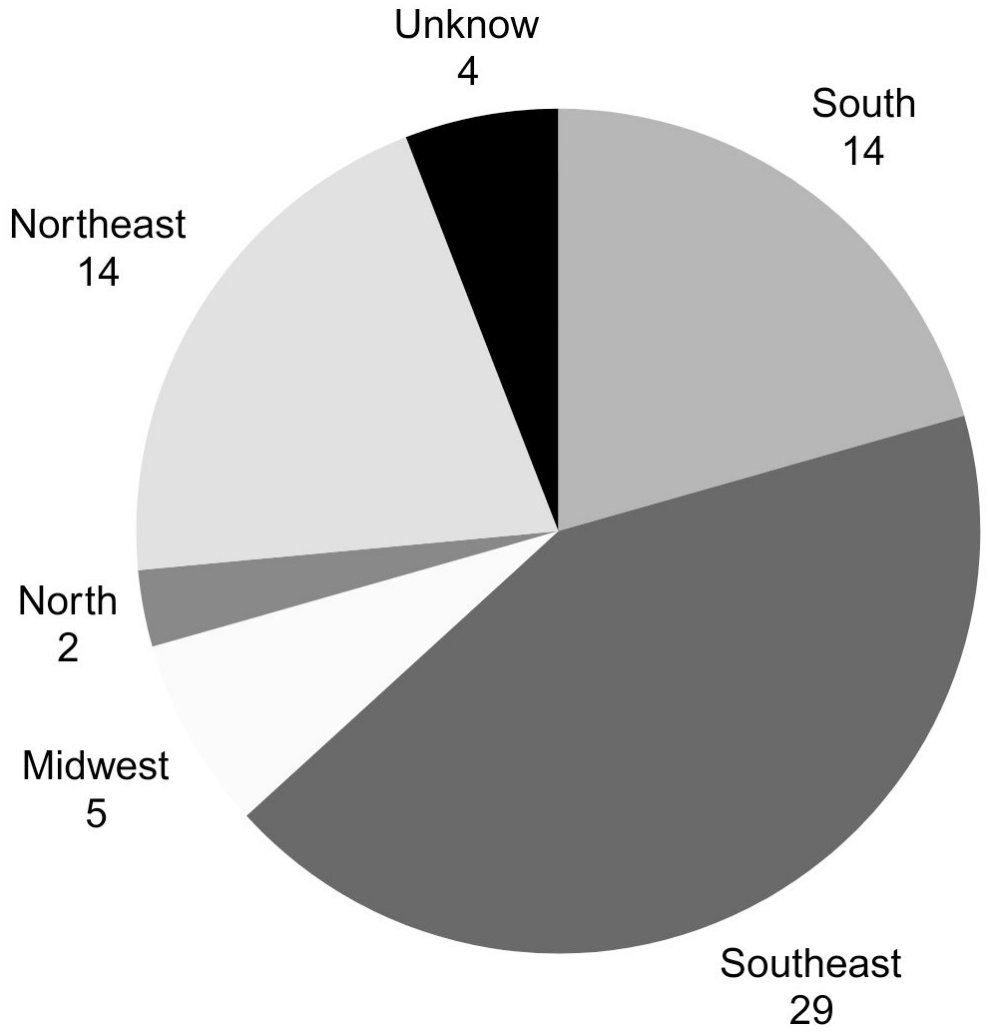
Distribution of patients by birth region in Brazil.

The association currently includes 126 individuals with CHI, and data from 68 individuals (38 males and 30 females) were collected after contacting all representatives to answer the questionnaire. These children are distributed across 19 of Brazil’s 26 states, in a proportionate manner relative to the country’s most populous regions. **Figure 1** illustrates the distribution according to the birth region of CHI children.

**Figure 2:**
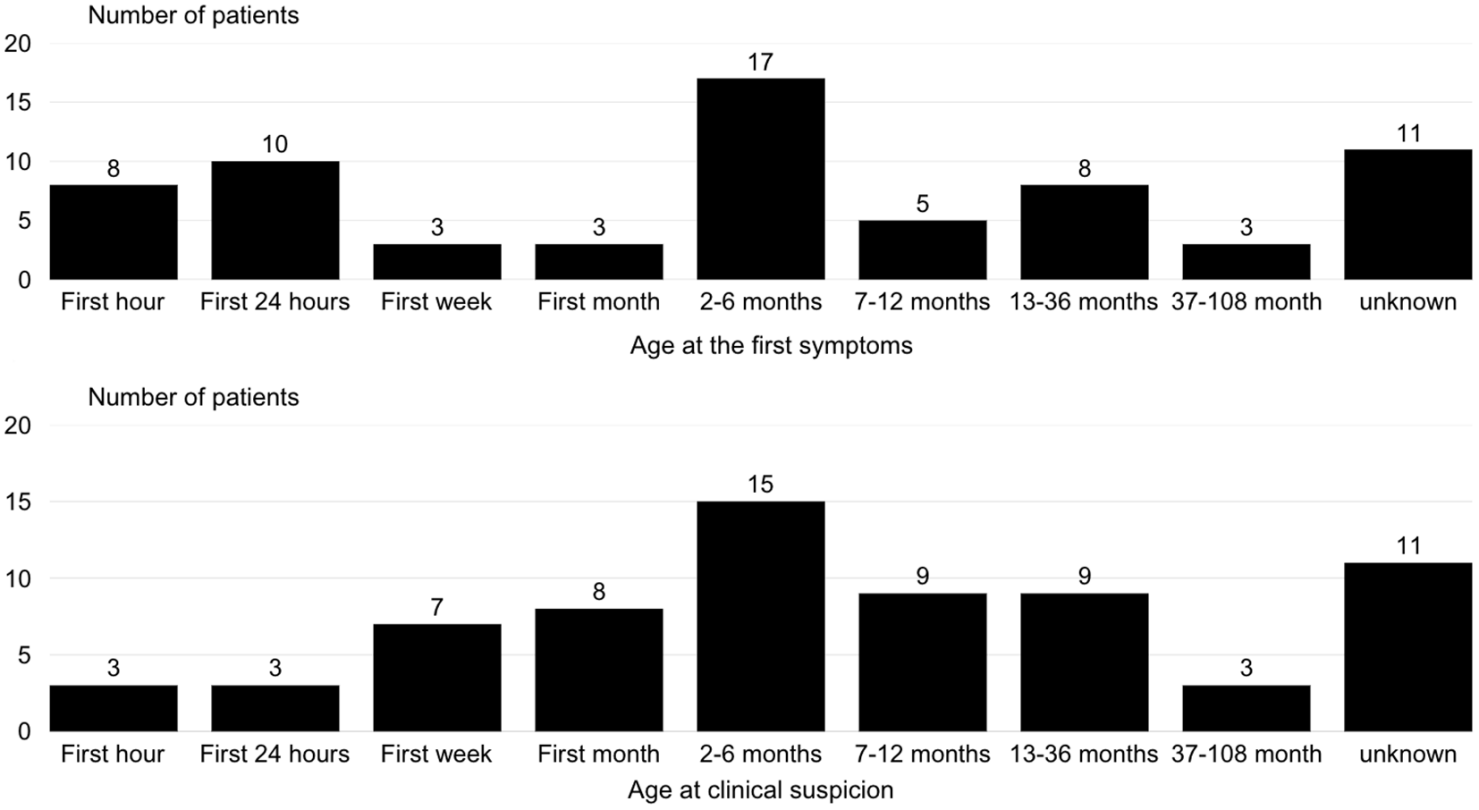
Distribution of age at first clinical signs and clinical suspicion in patients with congenital hyperinsulinism.

The initial symptoms occurred within the first 6 months of life in all the children. In 36 out of 68 cases, the diagnosis was also made before 6 months of age. **Figures 2a, b** describe the frequency of the first clinical signs and clinical suspicion by age group.

**Figure 3:**
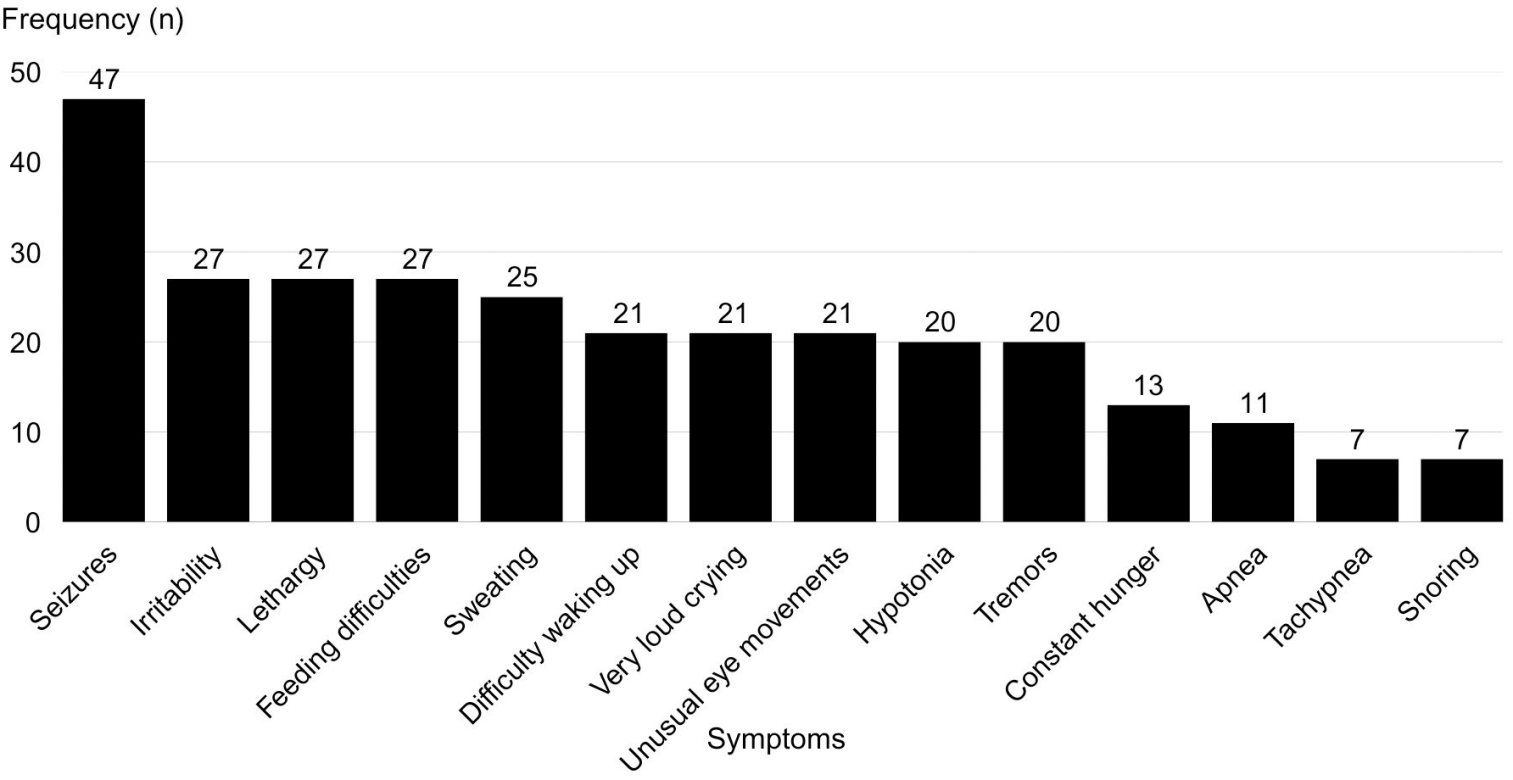
Frequency of reported symptoms associated with hypoglycemia in patients with congenital hyperinsulinism.

The most frequent symptoms and their occurrence rates are detailed in **Figure 3**. A total of 24 out of 68 patients initially received a misdiagnosis, such as epilepsy, transient hypoglycemia, or sepsis, before CHI was confirmed. Additionally, 57 out of 68 patients had been evaluated by three or more physicians before receiving the correct diagnosis.

Over three-quarters of the children were of appropriate weight for gestational age at birth.

Forty-three individuals underwent genetic testing, and pathogenic variants in *ABCC8* (n=5), *KCNJ11* (n=1), and *GLUD1* (n=1) were identified. No pathogenic variants were found in five individuals, while in the remaining participants reports were not available. 18F-DOPA PET-CT was performed in 4 out of 68 cases, all of whom had a paternally inherited recessive variant in one of the ATP-sensitive potassium channel-related genes.

A total of 13 individuals underwent pancreatectomy. After pancreatectomy, the histological form of CHI was determined as focal in 4 cases (the same cases that underwent 18F-DOPA PET-CT) and diffuse in 9 cases. In the remaining subjects who did not undergo pancreatectomy, the histological classification remained unknown.

Following diagnosis, 97% (66 out of 68) of the individuals required medical or surgical intervention. As of the time of data collection, two patients were able to manage their condition through dietary therapy alone.

At the time of the study, the most used medication was diazoxide alone (41/68), followed by octreotide alone (8/68) and lanreotide alone (2/68). In some cases, patients required a combination of medications. The following combinations were documented, each in one case: diazoxide and hydrochlorothiazide, diazoxide and growth hormone, diazoxide and octreotide, octreotide and sirolimus, and octreotide and lanreotide.

A total of 12 individuals were not using any medication at the time of the study, as all of them had undergone pancreatectomy. Only one patient required diazoxide after pancreatectomy.

Regarding nutritional support, 36 individuals reported exclusive oral feeding, while 11 received nutrition via nasogastric tube, 8 via enteral tube, and 13 required gastrostomy feeding.

In 30 out of 68 cases, caregivers reported developmental delays. Twenty-three patients exhibited motor developmental delays, 8 individuals had speech delays, and 5 reported learning difficulties in school. In 6 of these 30 cases, motor developmental delays were also associated with speech delays.

## Discussion

This study describes the clinical and epidemiological profile of congenital hyperinsulinism (CHI) in Brazil, highlighting the challenges in diagnosis and treatment. The findings confirm the complexity of early diagnosis, with 35.5% of patients initially receiving incorrect diagnoses, such as epilepsy and transient hypoglycemia. This diagnostic delay has also been reported in international studies, where many patients require consultations with multiple specialists before the disease is confirmed (6).

The frequency of symptoms identified in this study is consistent with the literature. Previous studies report that neonatal hypoglycemia, associated with symptoms such as lethargy and seizures, is the most common manifestation of CHI (1, 10). However, the present study emphasizes that despite early clinical manifestations, definitive diagnosis is often delayed, reinforcing the need for greater medical training to recognize the condition.

In a regional comparison, Brazil hosts a significantly larger population than neighboring countries. Despite this, access to diagnostic and treatment resources remains limited. Argentina, for example, offers molecular testing and 18F-DOPA PET-CT scans for CHI diagnosis, whereas PET-CT is not available in Brazil. The need for improved diagnostic strategies is further underscored by the high percentage of misdiagnosed patients and the fact that 83.5% required consultations with three or more physicians before receiving a correct diagnosis. These findings emphasize the importance of strengthening medical education and implementing standardized clinical protocols for early CHI recognition.

The identification of pathogenic variants in *ABCC8, KCNJ11*, and *GLUD1* in 16% of genetically tested patients supports the prevalence of these mutations as the primary genetic causes of CHI (5). However, the genetic testing rate in Brazil (63%) remains below ideal, limiting molecular diagnosis and individualized treatment. This limitation has also been observed in other Latin American countries, such as Argentina and Colombia with access to genetic testing and specialized imaging technologies (7).

Regarding treatment, diazoxide was the most frequently used medication, aligning with global practices (1). However, access to diazoxide is restricted in Brazil compared to Argentina and Colombia, where the medication is readily available. In Brazil, diazoxide is not registered by health authorities, requiring importation, which complicates access due to logistical and financial constraints. Although diazoxide is effective in most cases, a subset of patients remains unresponsive to the drug and requires alternative therapies, including somatostatin analogs and, in refractory cases, pancreatectomy. The necessity of surgical intervention in some patients highlights the severity of CHI in its extreme forms.

The study also shows the high morbidity associated with CHI, particularly when diagnosis and treatment are delayed. Forty-four percent of patients exhibited developmental delays; a percentage similar to reported by Ludwig et al. (3). This reinforces the importance of early intervention to mitigate long-term neurological consequences.

Overall, these findings highlight the urgent need to expand access to diagnostic tools, such as genetic sequencing and PET imaging, as well as specialized treatments. Additionally, they emphasize the importance of educational programs for healthcare professionals and caregivers to improve the recognition of CHI symptoms.

## Conclusion

This study detailed the clinical and epidemiological profile of CHI in Brazil, highlighting challenges in early diagnosis and access to treatment. The findings reinforce the need to raise medical awareness about CHI to ensure early recognition and prevent neurological complications.

A limitation of this study is that the data were obtained from caregiver reports rather than medical record reviews, which may introduce recall bias and inaccuracies in some information. However, this approach allowed for a comprehensive assessment of the disease’s impact on patients and their families, providing valuable insights into the challenges they face.

Given these findings, strengthening public policies to expand access to genetic testing and advanced therapies is essential, along with implementing national guidelines for CHI management.

Future multicenter studies, including medical record analysis and longitudinal follow-up, will be critical to deepening the understanding of CHI and optimizing its management in Brazil.

## Data Availability

The original contributions presented in the study are included in the article/supplementary material. Further inquiries can be directed to the corresponding author.

## References

[1] De Leon DD Arnoux JB Banerjee I Bergada I Bhatti T Conwell LS. International guidelines for the diagnosis and management of hyperinsulinism. Hormone Res Paediatrics. (2024) 97:279–98. doi: 10.1159/000531766 PMC11124746 37454648

[2] Lord K Radcliffe J Gallagher PR Adzick NS Stanley CA De León DD. High risk of diabetes and neurobehavioral deficits in individuals with surgically treated hyperinsulinism. J Clin Endocrinol Metab. (2015) 100:4133–9. doi: 10.1210/jc.2015-2539 PMC4702456 26327482

[3] Ludwig A Enke S Heindorf J Empting S Meissner T Mohnike K. Formal neurocognitive testing in 60 patients with congenital hyperinsulinism. Hormone Res Paediatrics. (2017) 88:220–8. doi: 10.1159/000481774 29151084

[4] De Franco E Flanagan SE Yau D Houghton JA Laver TW Ellard S. Update of variants identified in the pancreatic β-cell KATP channel genes *KCNJ11* and *ABCC8* in individuals with congenital hyperinsulinism and diabetes. Hum Mutat. (2020) 41:884–905. doi: 10.1002/humu.23987 32027066 PMC7187370

[5] Kapoor RR Flanagan SE Arya VB Shield JP Ellard S Hussain K. Clinical and molecular characterisation of 300 patients with congenital hyperinsulinism. Eur J Endocrinol. (2013) 168:557–64. doi: 10.1530/EJE-12-0673 PMC3599069 23345197

[6] Demirbilek H Hussain K. Congenital hyperinsulinism: diagnosis and treatment update. J Clin Res Pediatr Endocrinol. (2017) 9:69–87. doi: 10.4274/jcrpe2017.S007 29280746 PMC5790328

[7] Liberatore RD Monteiro ICM Pileggi FO Canesin WC Sbragia L. Congenital hyperinsulinism and surgical outcome in a single tertiary center in Brazil. Jornal Pediatria. (2024) 100:163–8. doi: 10.1016/j.jped.2023.09.005 PMC10943321 37866397

[8] Shuman C Kalish JM Weksberg R. Beckwith-wiedemann syndrome. In: Adam MP Feldman J Mirzaa GM Pagon RA Wallace SE Amemiya A, editors. GeneReviews. University of Washington, Seattle, Seattle (WA (2000). p. 1993–2025. doi: 10.1016/j.jped.2023.09.005 20301568

[9] Lapidus D De León DD Thornton PS Hood D Breit J Raskin J. The birth prevalence of congenital hyperinsulinism: A narrative review of the epidemiology of a rare disease. Hormone Res paediatrics. (2024), 1–8. doi: 10.1159/000539464 PMC12416878 38885633

[10] Yau D Laver TW Dastamani A Senniappan S Houghton JAL Shaikh G. Using referral rates for genetic testing to determine the incidence of a rare disease: The minimal incidence of congenital hyperinsulinism in the UK is 1 in 28,389. PLoS One. (2020) 15:e0228417. doi: 10.1371/journal.pone.0228417 32027664 PMC7004321

[11] Arya VB Senniappan S Demirbilek H Alam S Flanagan SE Ellard S. Pancreatic endocrine and exocrine function in children following near-total pancreatectomy for diffuse congenital hyperinsulinism. PLoS One. (2014) 9:e98054. doi: 10.1371/journal.pone.0098054 24840042 PMC4026387

[12] Pasquini TLS Mesfin M Schmitt J Raskin J. Global registries in congenital hyperinsulinism. Front Endocrinol. (2022) 13:876903. doi: 10.3389/fendo.2022.876903 PMC9201947 35721728

